# Level and Prevalence of Spin in Published Cardiovascular Randomized Clinical Trial Reports With Statistically Nonsignificant Primary Outcomes: A Systematic Review

**DOI:** 10.1001/jamanetworkopen.2019.2622

**Published:** 2019-05-03

**Authors:** Muhammad Shahzeb Khan, Noman Lateef, Tariq Jamal Siddiqi, Karim Abdur Rehman, Saed Alnaimat, Safi U. Khan, Haris Riaz, M. Hassan Murad, John Mandrola, Rami Doukky, Richard A. Krasuski

**Affiliations:** 1 Department of Internal Medicine, John H. Stroger, Jr. Hospital of Cook County, Chicago, Illinois; 2 Department of Internal Medicine, Creighton University Medical Center, Omaha, Nebraska; 3 Department of Internal Medicine, Dow University of Health Sciences, Karachi, Pakistan; 4 Department of Cardiovascular Medicine, Cleveland Clinic, Cleveland, Ohio; 5 Department of Internal Medicine, University of Iowa, Iowa City; 6 Department of Internal Medicine, Robert Packer Hospital, Sayre, Pennsylvania; 7 Evidence-Based Practice Center, Mayo Clinic, Rochester, Minnesota; 8 Department of Cardiovascular Medicine, Baptist Health Louisville, Louisville, Kentucky; 9 Department of Cardiovascular Medicine, John H. Stroger, Jr. Hospital of Cook County, Chicago, Illinois; 10 Department of Cardiovascular Medicine, Duke University Health System, Durham, North Carolina

## Abstract

**Importance:**

Clinical researchers are obligated to present results objectively and accurately to ensure readers are not misled. In studies in which primary end points are not statistically significant, placing a spin, defined as the manipulation of language to potentially mislead readers from the likely truth of the results, can distract the reader and lead to misinterpretation and misapplication of the findings.

**Objective:**

To determine the level and prevalence of spin in published reports of cardiovascular randomized clinical trial (RCT) reports.

**Data Source:**

MEDLINE was searched from January 1, 2015, to December 31, 2017, using the Cochrane highly sensitive search strategy.

**Study Selection:**

Inclusion criteria were parallel-group RCTs published from January 1, 2015, to December 31, 2017 in 1 of 6 high-impact journals (*New England Journal of Medicine*, *The Lancet*, *JAMA*, *European Heart Journal*, *Circulation*, and *Journal of the American College of Cardiology*) with primary outcomes that were not statistically significant were included in the analysis.

**Data Extraction and Synthesis:**

Analysis began in August 2018. Data were extracted and verified by 2 independent investigators using a standard collection form. In cases of disagreement between the 2 investigators, a third investigators served as arbitrator.

**Main Outcomes and Measures:**

The classifications of spin type, severity, and extent were determined according to predefined criteria. Primary clinical outcomes were divided into safety of treatment, efficacy of treatment, and both.

**Results:**

Of 587 studies identified, 93 RCT reports (15.8%) met inclusion criteria. Spin was identified in 53 abstracts (57%; 95% CI, 47%-67%) and 62 main texts of published articles (67%; 95% CI, 57%-75%). Ten reports (11%; 95% CI, 6%-19%) had spin in the title, 35 reports (38%; 95% CI, 28%-48%) had spin in the results section, and 50 reports (54%; 95% CI, 44%-64%) had spin in the conclusions. Among the abstracts, spin was observed in 38 results sections (41%; 95% CI, 31%-51%) and 45 conclusions sections (48%; 95% CI, 38%-58%).

**Conclusions and Relevance:**

This study suggests that in reports of cardiovascular RCTs with statistically nonsignificant primary outcomes, investigators often manipulate the language of the report to detract from the neutral primary outcomes. To best apply evidence to patient care, consumers of cardiovascular research should be aware that peer review does not always preclude the use of misleading language in scientific articles.

## Key Points

**Question:**

Do authors of cardiovascular randomized clinical trials present statistically nonsignificant primary outcomes accurately and objectively?

**Findings:**

In this systematic review that included 93 reports of randomized clinical trials from 6 high-impact journals, positive spin of statistically nonsignificant primary outcomes was found in 57% of abstracts and 67% of main text of the published articles.

**Meaning:**

Despite peer review, manipulation of language in the cardiovascular literature is common and may have implications for scientific integrity, patient care, peer review, and medical progress.

## Teaser

This systematic review examines the level and prevalence of spin in presenting findings that are not statistically significant in published reports of cardiovascular randomized clinical trials (RCTs).

## Introduction

Randomized clinical trials (RCTs) are considered the criterion standard for determining efficacy of an intervention. Methodologically rigorous RCTs generate the highest certainty of evidence and form the basis of clinical guidelines.^1^ Evidence-based practice depends on accurate presentation of a trial’s results. Journal editors allow authors of scientific articles broad latitude in the use of language when reporting their study, which may subconsciously or consciously shape the impression of their results for readers.

*Spin* is defined as the manipulation of language to potentially mislead readers from the likely truth of the results.^2,3,4^ Boutron et al^5^ developed a technique to identify and classify spin in RCT reports. Their approach focused on RCTs reporting statistically nonsignificant primary outcomes because the interpretation of these results is more likely to be subject to prior beliefs of effectiveness, leading to potential bias in reporting.^6^ Subsequent studies have used similar approaches to systematically assess the explicit presentation of nonsignificant results in trial reports in various subspecialties.^7,8^ Although previous articles have briefly highlighted distorted presentation and interpretation of results in the cardiovascular (CV) literature,^9,10^ to our knowledge, these strategies have not been systematically identified and evaluated. Such reporting bias may lead to inaccurate estimation of the benefit of CV interventions and affect patient care. We aimed to assess the prevalence and extent of spin in a cohort of CV RCT reports.

## Methods

This systematic review is reported following the Preferred Reporting Items for Systematic Reviews and Meta-analyses (PRISMA) reporting guideline and the Strengthening the Reporting of Observational Studies in Epidemiology (STROBE) reporting guideline for cohort studies.^11,12^

### Search, Eligibility, and Selection of Studies

We searched MEDLINE using the Cochrane highly sensitive search strategy^13^ for CV RCTs published from January 1, 2015, to December 31, 2017, in 6 high-impact journals (*New England Journal of Medicine*, *The Lancet*, *JAMA*, *European Heart Journal*, *Circulation*, and *Journal of the American College of Cardiology*). A detailed search strategy is provided in the Appendix in the Supplement. We included only RCTs with parallel groups (defined as studies in which each participant is randomized to 1 of the intervention arms) and clearly identified primary outcomes (ie, distinctly mentioned in the trial as the primary study objective) that were not statistically significant (ie, *P* ≥ .05).

We excluded pilot studies, brief communications, research letters, factorial and split-body designs, cluster trials, equivalence or noninferiority trials, crossover trials, multigroup trials, and phase 1 or 2 trials. Trials that exclusively focused on economic evaluations and diagnostic test accuracy were also excluded. The definitions and strategies for the genre of trials reflect those used by Chan and Altman.^14^

The preliminary search identified 2473 RCT reports cited in PubMed, which were then transferred to EndNote (Clarivate Analytics). The titles and abstracts of the identified studies were then screened to exclude irrelevant studies. Full-text studies were subsequently obtained and evaluated for the remaining 1166 reports. After assessing for relevance, 587 studies were included. The screening process was carried out by 2 independent reviewers (N.L. and T.J.S.), and a third reviewer (M.S.K.) was consulted in the event of discrepancies.

### Data Extraction From Selected Studies

For each selected RCT report, we extracted 2016 journal impact factor, number of citations in PubMed until August 2018, source of funding, intervention in the active and control groups, primary clinical outcome, and presence or absence of conflict of interest of the first and last authors. Presence of conflict of interest for the first and last authors was categorized as yes or no. Two reviewers (N.L. and T.J.S.) extracted all relevant data onto a standardized data collection form and then independently read the title, abstract and methods, results, discussion, and conclusions sections to identify the type, severity, and extent of spin, if any. Any disagreements were resolved through discussion. When a consensus could not be reached, a third author (M.S.K.) arbitrated. The classifications of spin type, severity, and extent were based on the article by Boutron et al.^5^ All the authors were briefed about these criteria before data collection and all unanimously agreed to its application.

The κ coefficient is a measure of the extent of agreement between the 2 independent investigators and was calculated using the methodology outlined by Landis and Koch.^15^ The frequency of disagreements and agreements between the reviewers were computed in the Kappa Calculator (Statistics Solutions),^16^ and the κ statistics were determined for each outcome. There was substantial agreement in reproducibility for presence of spin, with κ = 0.77 (95% CI, 0.52-0.91) in the main text conclusions section, κ = 0.73 (95% CI, 0.41-0.87) in the abstract conclusions section, κ =  0.79 (95% CI, 0.53-0.90) in the main text results section, κ = 0.64 (95% CI, 0.42-0.79) in the abstract results section, κ = 0.75 (95% CI, 0.57-0.96) in the discussion section, and κ = 0.65 (95% CI, 0.57-0.89) for presence of spin in the title.

Primary clinical outcomes were divided into 3 categories: safety of treatment, efficacy of treatment, and both. Randomized clinical trials in which the primary outcome was related to measurement of adverse events from a treatment were classified under safety of treatment. Trials in which the primary outcome was related to measure of effectiveness of a drug (eg, reduction in mortality or glycemic or blood pressure levels) were classified under efficacy of treatment. We also divided the end point by type: dichotomous, other, or both.

### Definition and Classification of Spin

Following the methods outlined by Boutron et al,^5^ we defined *spin* as the “use of specific reporting strategies, from whatever motive, to highlight that the experimental treatment is beneficial, despite a statistically nonsignificant difference for the primary outcome [ie, inappropriate use of causal language], or to distract the reader from statistically nonsignificant results [ie, to focus on a statistically significant secondary result].”^5^ Using a prespecified spin classification scheme, the following sections of each trial were scrutinized to determine whether authors had used spin: the title, the results and conclusions sections of the abstract, and the results, discussion, and conclusions sections of the main text.^6^ We then identified which of 3 specific spin strategies had been used: (1) authors pivoted on statistically significant secondary results in the form of focus on within-group comparison, secondary outcomes, subgroup, or per-protocol analyses; (2) authors interpreted statistically nonsignificant results of the primary outcomes to show treatment equivalence or to rule out an adverse event; and (3) authors emphasized the beneficial effect of the treatment with or without acknowledging the statistically nonsignificant primary outcome. Strategies of spin that could not be classified under 1 of the 3 schemes were systematically recorded as *other*.

### Level of Spin in Conclusions

We also assessed the level of spin in the abstract and main text conclusions section. We classified the level of spin as high, moderate, or low, with *none* acting as a default category. High spin was defined as no acknowledgment of the statistically nonsignificant primary outcome, no uncertainty in the framing, and no recommendations for further trials. Moderate spin was defined as no acknowledgment of the nonsignificant primary outcome but some uncertainty in the framing or the presence of recommendations for further trials. Low spin was defined as acknowledgment of the nonsignificant primary outcome or uncertainty in the framing and presence of recommendations for further trials. The purpose of calculating level of spin was to assess the heterogeneity of reporting of spin in the conclusions section.

### Extent of Spin

We calculated the extent of spin across the whole of each report, defined as number of sections with spin in both the abstract and main text. Spin in the title was excluded from this calculation. We used 4 categories to classify the extent of spin: (1) spin in 1 section other than the conclusions section; (2) spin in the conclusions section only; (3) spin in 2 sections, not including the abstract; and (4) spin in all sections.

### Statistical Analysis

We calculated the number and percentage of reports with 95% CIs for categorical variables and medians with interquartile ranges for continuous variables. The χ^2^ linear-by-linear association test was conducted to assess the association of level of spin in the abstract or text with first or last author conflict of interest. The Spearman ρ correlation test was performed to evaluate any correlation between spin in the text or abstract and the citations per year of each report. Finally, the Kruskal-Wallis test with Dunn post hoc method was used to evaluate whether spin was associated more with a certain type of end point, type of primary outcome, type of experimental treatment (eg, drug, surgical operation or procedure, or device), comparator (eg, placebo, drug, or device), subspecialty, funding source, or journal. All statistical analyses were performed using SPSS Statistics version 23.0 (IBM). *P* values were 2-tailed, and a *P* value less than .05 was considered statistically significant.

## Results

### General Characteristics of Selected Studies

Of 587 studies included in the sample, we identified 229 parallel-group RCTs with clearly identified primary outcomes. Of these, 93 studies reported statistically nonsignificant results for their primary outcome (Figure). We identified 25 studies (27%; 95% CI, 19%-37%) funded solely by for-profit sources and 37 studies (40%; 95% CI, 30%-50%) funded solely by nonprofit sources. Detailed characteristics of the included RCTs are outlined in Table 1.

**Figure.**
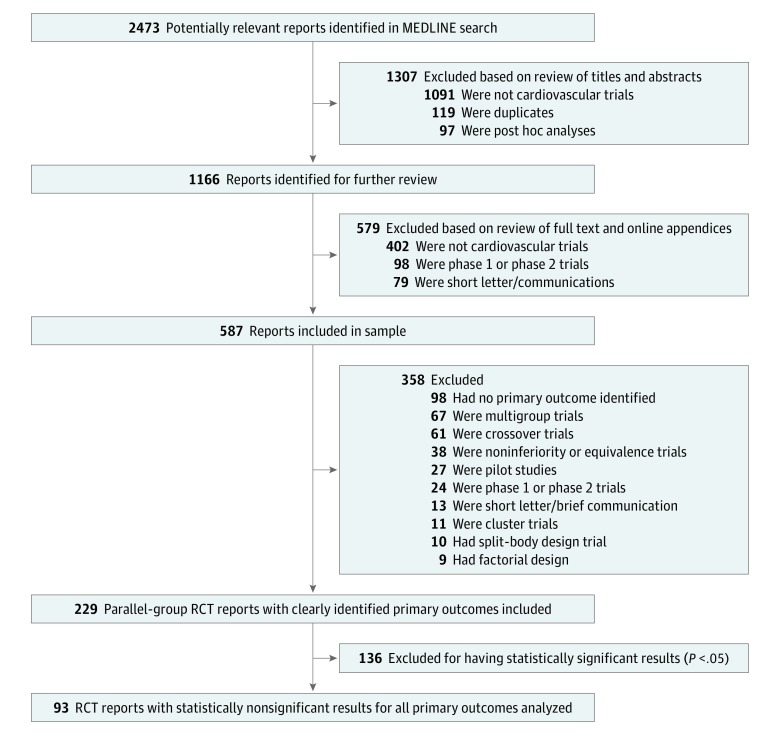
PRISMA Flowchart Outlining the Search Strategy RCT indicates randomized clinical trial.

**Table 1.** Trial Characteristics

Characteristic	No. (%) [95% CI, %]
Total, No.	93
End points	
Safety of treatment	3 (3) [1.1-9.1]
Efficacy of treatment	84 (90) [82.6-94.8]
Safety and efficacy	6 (6) [3.0-13.3]
Primary outcome	
Binary	38 (41) [31.4-51.0]
Other	52 (56) [45.8-65.6]
Binary and other	3 (3) [1.1-9.1]
Sample size, median (IQR) [range]	1002 (303-2539) [48-18201]
Journal impact factor, median (IQR) [range]	32.3 (20-48) [19-72]
No. of citations up to May 2018, median (IQR) [range]	7 (4-17) [0-86]
Experimental treatment	
Drug	40 (43) [33.4-53.1]
Surgical operation or procedure	25 (27) [18.9-36.7]
Device	7 (8) [3.7-14.7]
Participative intervention (eg, rehabilitation or education)	4 (4) [1.6-10.5]
Other	17 (18) [11.7-27.3]
Comparator	
Placebo	22 (22) [16.2-33.2]
Device	1 (1) [0.2-5.9]
Standard care	28 (30) [21.7-40.1]
Drug	12 (13) [7.5-21.2]
Surgical operation/procedure	23 (25) [17.1-34.4]
Participative intervention (eg, rehabilitation or education)	2 (2) [0.6-7.5]
Other	5 (5) [2.3-12.0]
Funding source	
None	0 (0) [0-0.4]
For profit	25 (27) [18.9-36.7]
Nonprofit	37 (40) [30.4-50.0]
For profit and nonprofit	19 (20) [13.5-29.7]
None reported	12 (13) [7.5-21.2]

Abbreviation: IQR, interquartile range.

### Overview

Table 2 outlines the frequency of spin observed in each section of the abstracts and texts. Spin was observed in the titles of 10 reports (11%; 95% CI, 6%-19%). In the abstracts, we identified spin in 38 results sections (41%; 95% CI, 31%-51%) and in 45 conclusions sections (48%; 95% CI, 38%-58%). In the main texts, 35 results sections (38%; 95% CI, 28%-48%) and 35 discussion sections (38%; 95% CI, 28%-48%) showed spin. Moreover, 50 of the main text conclusions sections (54%; 95% CI, 44%-64%) included spin.

**Table 2.** Classification of Spin in the Title, Abstract, and Main Text of Randomized Clinical Trials

Type of Spin	No. (%) [95% CI]	
	Abstract (n = 93)	Main Text (n = 93)
Title	NA	10 (11) [5.9-18.7]
Results section	38 (41) [31.4-51.0]	35 (38) [28.4-47.8]
Focusing on statistically significant within-group comparison	8 (9) [4.4-16.1]	8 (9) [4.4-16.0]
Focusing on statistically significant secondary outcomes	13 (14) [8.3-22.5]	11 (12) [6.7-19.9]
Focusing on statistically significant subgroup analyses	10 (11) [5.9-18.7]	8 (9) [4.4-16.0]
Focusing on statistically significant modified population of analyses (eg, per-protocol analyses)	0 (0) [0-3.9]	0 (0) [0-3.9]
Focusing on statistically significant within-group and between-group comparisons for secondary outcomes	1 (1) [0.2-5.8]	1 (1) [0.2-5.8]
Other	6 (6) [3.0-13.4]	7 (8) [3.7-14.7]
Discussion section	NA	35 (38) [28.4-47.8]
Reporting of statistically nonsignificant outcome as if the trial were an equivalence trial	NA	5 (5) [2.3-12.0]
Focusing on statistically significant secondary outcomes	NA	7 (8) [3.7-14.7]
Focusing on statistically significant subgroup analyses	NA	6 (6) [3.0-13.4]
Focusing on statistically significant modified population of analyses (eg, per-protocol analyses)	NA	0 (0) [0-3.9]
Focusing on overall within-group improvement	NA	7 (8) [3.7-14.7]
Ruling out adverse event	NA	1 (1) [0.2-5.8]
Other	NA	9 (10) [5.2-17.4]
Conclusions section	45 (48) [38.5-58.4]	50 (54) [43.7-63.5]
Focusing only on treatment effectiveness	17 (18) [11.7-27.3]	16 (17) [10.9-26.1]
Claiming equivalence for statistically nonsignificant results	9 (10) [5.2-17.4]	6 (6) [3.0-13.4]
Claiming efficacy with no consideration of statistically nonsignificant primary outcome	4 (4) [1.7-10.5]	7 (8) [3.7-14.7]
Focusing only on statistically significant results	4 (4) [1.7-10.5]	3 (3) [1.1-9.1]
Acknowledging statistically nonsignificant results for the primary outcome but emphasizing beneficial effect of treatment	2 (2) [0.6-7.5]	6 (6) [3.0-13.4]
Acknowledging statistically nonsignificant results for the primary outcome but emphasizing other statistically significant results	9 (10) [5.2-17.4]	12 (13) [7.1-21.8]
Other spin in conclusions section, No. (%)	13 (14)	13 (14)
Conclusion ruling out an adverse event on statistically nonsignificant results	2 (2) [0.6-7.5]	0 (0) [0-3.9]
Conclusion focusing on within-group assessment (both treatments are effective or treatment administered in both groups is effective [eg, add-on studies])	1 (1) [0.2-5.8]	1 (1) [0.2-5.8]
Recommending to use the treatment	1 (1) [0.2-5.8]	3 (3) [1.1-9.1]
Focusing on another objective	2 (2) [0.6-7.5]	4 (4) [1.7-10.5]
Comparing with placebo group of another trial	0 (0) [0-3.9]	0 (0) [0-3.9]
Reporting statistically nonsignificant subgroup results as beneficial	0 (0) [0-3.9]	1 (1) [0.2-5.8]
Other	7 (8) [3.7-14.7]	4 (4) [1.7-10.5]

Abbreviation: NA, not applicable.

### Spin Strategies

The strategies of spin in each section of the abstract and full text are presented in detail in Table 2. In the abstracts, spin in the results sections was found in 13 reports (14%; 95% CI, 8%-22%) focusing on statistically significant secondary outcomes and in 10 reports (11%; 95% CI, 6%-19%) focusing on statistically significant subgroup analyses, whereas 17 reports (18%; 95% CI, 12%-27%) that used spin in the conclusions section mainly focused on reporting treatment effectiveness. In the main texts, spin in the results section included 11 reports (12%; 95% CI, 7%-20%) presenting statistically significant secondary outcomes and in the conclusions section in 16 reports (17%; 95% CI, 11%-26%) focusing on treatment effectiveness.

### Extent of Spin

The extent of spin varied among our cohort (Table 3). Overall, 53 abstracts (57%; 95% CI, 47%-67%) and 62 main texts (67%; 95% CI, 57%-75%) had spin in at least 1 section. Moreover, 26 abstracts (28%; 95% CI, 20%-38%) and 18 main texts (19%; 95% CI, 12%-29%) had spin in all of their sections.

**Table 3.** Extent and Level of Spin in the Conclusions Sections of Randomized Clinical Trials

Spin	No. (%) [95% CI]	
	Abstract (n = 93)	Main Text (n = 93)
Extent of spin		
None	40 (43) [33.4-53.1]	31 (33) [24.6-43.4]
1 Section, other than conclusions	11 (12) [6.7-19.9]	12 (13) [7.5-21.2]
Conclusions only	16 (17) [10.9-26.1]	13 (14) [8.3-22.7]
2 Sections	NA	19 (20) [13.5-29.7]
All sections	26 (28) [19.9-37.8]	18 (19) [12.2-29.1]
Level of spin in conclusions section		
None	52 (56) [45.8-65.6]	44 (47) [37.5-57.4]
Low^a^	30 (32) [23.6-42.3]	36 (39) [29.5-48.9]
Moderate^b^	6 (6) [3.0-13.4]	5 (5) [2.3-12.0]
High^c^	5 (5) [2.3-12.0]	8 (9) [4.4-16.0]

Abbreviation: NA, not applicable.

^a^ Low spin was defined as acknowledgment of the nonsignificant primary outcome or uncertainty in the framing and presence of recommendations for further trials.

^b^ Moderate spin was defined as no acknowledgment of the nonsignificant primary outcome but some uncertainty in the framing or the presence of recommendations for further trials.

^c^ High spin was defined as no acknowledgment of the statistically nonsignificant primary outcomes, no uncertainty in the framing, and no recommendations for further trials.

### Level of Spin in Conclusions

Table 3 outlines the level of spin in the conclusions sections of the abstract and main text. About half the conclusions sections included spin, with 41 abstracts (44%; 95% CI, 34%-54%) and 49 full texts (53%; 95% CI, 43%-62%) containing some level of spin. In reports where spin was identified in the conclusions, it was mostly low-level spin.

### Correlations and Associations

Conflicts of interest disclosures of the first authors (abstract, χ^2^ = 0.215, *P* = .64; full text, χ^2^ = 0.003, *P* = .96) and last authors (abstract, χ^2^ = 1.675, *P* = .20; full text, χ^2^ = 2.644, *P* = .10) did not correlate with spin. A modest but significant negative correlation was present between citations per year and the level of spin in the abstract (*ρ* = −0.20; *P* = .03) and main text (*ρ* = −0.3; *P* = .049).

In the main text of the articles, we found that spin was not significantly associated with CV subspecialty, industry funding, or type of journal. Articles in some journals were found to have more spin than those in other journals. However, given the relatively small number of articles from each journal, this finding should be considered exploratory and must be viewed with caution. Table 4 details these associations.

**Table 4.** Elements Associated With Level of Spin in Cardiovascular Randomized Clinical Trial Reports

Element	Abstract		Main Text	
	χ^2^ (*P* Value)^a^	Interpretation^b^	χ^2^ (*P* Value)^a^	Interpretation^b^
End point	4.285 (.12)	No significant association	10.463 (.005)	Level of spin varied according to type of end point; studies that measured efficacy as their primary outcome were significantly more likely to use spin in their main text when compared with studies measuring safety as their primary outcome (*P* = .02)
Primary outcome	6.033 (.049)	Level of spin varied according to type of primary outcome; studies with nonbinary primary outcomes were significantly more likely to use spin in the abstract compared with studies with binary primary outcomes (*P* = .048)	3.060 (.28)	No significant association
Experimental arm	3.965 (.41)	No significant association	1.345 (.85)	No significant association
Comparator arm	15.266 (.02)	Level of spin varied according to type of comparator arm; studies were more likely to have spin in the abstract when the comparator was a drug compared with studies with standard care as the comparator arm (*P* = .03)	11.877 (.07)	No significant association
Subspecialty	5.363 (.50)	No significant association	2.462 (.87)	No significant association
Funding	12.011 (.65)	No significant association	5.101 (.34)	No significant association
Journal	19.681 (.001)	Level of spin varied according to journal; level of spin in the abstract was associated with the journal (*P* = .001); specifically, studies published in *JACC* were more likely to have spin in their abstract compared with studies published in *JAMA* (*P* = .01) and *NEJM* (*P* = .02)	10.115 (.72)	No significant association

Abbreviations: *JACC*, *Journal of the American College of Cardiology*; *NEJM*, *New England Journal of Medicine*.

^a^ *P* value was found from the Kruskal-Wallis test.

^b^ *P* value was found from the Dunn post hoc method.

## Discussion

In RCTs with statistically nonsignificant primary outcomes published in high-impact CV journals, we found considerable manipulation of language in both the abstracts and the full texts of the RCT reports. This occurred in RCTs that received industry or public funding. Our results align well with findings of spin in reports from other medical areas.^6,10^

When we found spin in the results and discussion sections of studies with nonsignificant primary efficacy outcomes, authors tended to focus on statistically significant secondary end points, within-group analyses, and subgroup analyses. In some cases of spin, within-group comparisons showed statistical significance in the experimental arm but not in the comparator, which was interpreted as a potential benefit of the treatment. Other ways authors used spin were to report lack of harm from the safety data without highlighting the statistically nonsignificant efficacy result, to focus on effectiveness of both treatment arms when a statistically significant change was seen from baseline for each group, or to use both these methods to spin statistically nonsignificant results.

Unlike previous studies on spin, we did not observe an association of the level of spin with conflicts of interest disclosures from the first and last authors.^17,18^ More specifically, industry-funded research had a lower proportion of spin than nonprofit-funded research. This is important because it goes against a commonly expressed view that industry funding may have a direct or indirect effect on an investigator to explicitly describe nonsignificant results.^19,20,21,22^ However, a 2009 report^23^ highlighted high rates of underreporting of financial conflicts of interest by investigators. It is possible that researchers who are prone to spin may intentionally or unintentionally underreport their financial relationships.

We can only speculate on the reason authors use positive spin. Incentives likely play a role. Publication in high-impact journals fosters career advancement and future grant funding. A 2014 study^24^ on publication bias has shown that positive findings are more likely to be published in higher-impact journals. Authors may naturally tend to accentuate a positive approach to their results. We believe spin may decrease if neutral results were as likely to be published.

In some circumstances, additional spin beyond what is actually in the report may occur when it is published. For example, an accompanying press release by the journal, the authors’ institution(s), or the funding agency may focus on any positive aspects without mentioning the primary outcome. Furthermore, in some instances the funding agency may employ media coaches to teach investigators how to stay on message.

These observations have significant implications for the integrity of clinical science, the translation of clinical evidence at the bedside, peer review, and the rate of medical progress. Manipulation of language to distort findings may also lead to further public distrust in science. For example, it is estimated that around 5% to 10% of individuals have strong antivaccination beliefs.^25^ Among these individuals, lack of trust in health professionals or in public health institutions can be pivotal in making decisions about vaccinating their infants.^26^

Our findings support efforts to improve higher-quality reporting of scientific studies.^27^ The high prevalence of manipulation of language in the literature has great importance for consumers of medical evidence, which, given the freedom of information in the digital age, affects both patients as well as clinicians. Readers of medical evidence should be aware of these rhetorical techniques in science publications.^28,29,30^ More robust editorial and peer review may help reduce the level of spin. Finally, we propose that medical progress might move faster and waste fewer resources if authors were able to publish neutral findings in higher-impact journals.^31^ Knowing what does not work is also an important part of scientific discovery.

### Limitations

Our review has limitations that need to be considered when interpreting the results. First, although our study cohort is a representative sample of high-impact RCT reports, our search was limited to a short period in select journals. Second, we only selected studies with clearly defined primary outcomes and excluded studies without a clearly specified primary outcome, as the latter would not allow us to identify spin. Third, we were not able to assess from the data available which spin strategies were more successful, and neither were we able to conclude why spin occurs in medical research. Utmost efforts were made to avoid bias by following strict criteria for identification and assessment of spin by 2 independent reviewers. However, the presence of subjective bias cannot be completely excluded. Furthermore, we were not able to evaluate the effect of spin on peer reviewers, editors, and health care professionals on the receiving end. While this study is unique in the CV literature, to our knowledge, several unanswered questions arise from our findings, paving the way for future research to examine the mechanisms of spin and implications of trial reporting in the field of CV medicine.

## Conclusions

Our findings show that in approximately 67% of CV RCT reports, the reporting and interpretation of outcomes is inconsistent with actual results in at least 1 section of the article. Consumers of CV research should become familiar with the principles of evidence-based medicine to appraise and appropriately apply trial evidence.

## Notes

Supplement. **eAppendix.** MEDLINE detailed search strategy
