# Supplementary material for: Level and Prevalence of Spin in Published Cardiovascular Randomized Clinical Trial Reports With Statistically Nonsignificant Primary Outcomes: A Systematic Review
Source: JAMA Netw Open. 2019 May 3;2(5):e192622. doi: 10.1001/jamanetworkopen.2019.2622 (PMC6503494; doi:10.1001/jamanetworkopen.2019.2622)
Supplement: Supplement. — eAppendix. MEDLINE detailed search strategy [file jamanetwopen-2-e192622-s001.pdf]

Supplementary Online Content

Khan MS, Lateef N, Siddiqi TJ, et al. Level and prevalence of spin in published cardiovascular randomized clinical trial reports with statistically nonsignificant primary outcomes: a systematic review. *JAMA Netw Open*. 2019;2(5):e192622. doi:10.1001/jamanetworkopen.2019.2622

eAppendix. MEDLINE detailed search strategy

This supplementary material has been provided by the authors to give readers additional information about their work.

© 2019 Khan MS et al. *JAMA Network Open.*

eAppendix. MEDLINE detailed search strategy.

(randomized controlled trial [pt] OR controlled clinical trial [pt] OR randomized controlled trials [mh] OR random allocation [mh] OR double-blind method [mh] OR single-blind method [mh] OR clinical trial [pt] OR clinical trials [mh] OR (“clinical trial [tw]) OR ((singl* [tw] OR doubl* [tw] OR trebl* [tw] OR tripl* [tw]) AND (mask* [tw] OR blind*[tw])) OR (“latin square” [tw]) OR placebos [mh] OR placebo* [tw] Or random* [tw] OR research design [mh:noexp] OR comparative study [mh] OR evaluation studies [mh] OR follow-up studies [mh] OR prospective studies [mh] OR cross-over studies [mh] OR control* [tw] OR prospective* [tw] OR volunteer* [tw]) NOT (animal [mh] NOT human [mh])

© 2019 Khan MS et al. *JAMA Network Open.*
